# BOLD cardiovascular magnetic resonance at 3.0 tesla in myocardial ischemia

**DOI:** 10.1186/1532-429X-12-54

**Published:** 2010-09-22

**Authors:** Robert Manka, Ingo Paetsch, Bernhard Schnackenburg, Rolf Gebker, Eckart Fleck, Cosima Jahnke

**Affiliations:** 1 Department of Internal Medicine/Cardiology, German Heart Institute, Berlin, Germany; 2 Department of Cardiology, University Hospital RWTH Aachen, Germany; 3 Philips Clinical Sciences, Hamburg, Germany

## Abstract

**Background:**

The purpose of this study was to determine the ability of Blood Oxygen Level Dependent (BOLD) cardiovascular magnetic resonance (CMR) to detect stress-inducible myocardial ischemic reactions in the presence of angiographically significant coronary artery disease (CAD).

**Methods:**

Forty-six patients (34 men; age 65 ± 9 years,) with suspected or known coronary artery disease underwent CMR at 3Tesla prior to clinically indicated invasive coronary angiography. BOLD CMR was performed in 3 short axis slices of the heart at rest and during adenosine stress (140 μg/kg/min) followed by late gadolinium enhancement (LGE) imaging. In all 16 standard myocardial segments, T2* values were derived at rest and under adenosine stress. Quantitative coronary angiography served as the standard of reference and defined normal myocardial segments (i.e. all 16 segments in patients without any CAD), ischemic segments (i.e. supplied by a coronary artery with ≥50% luminal narrowing) and non-ischemic segments (i.e. supplied by a non-significantly stenosed coronary artery in patients with significant CAD).

**Results:**

Coronary angiography demonstrated significant CAD in 23 patients. BOLD CMR at rest revealed significantly lower T2* values for ischemic segments (26.7 ± 11.6 ms) compared to normal (31.9 ± 11.9 ms; p < 0.0001) and non-ischemic segments (31.2 ± 12.2 ms; p = 0.0003). Under adenosine stress T2* values increased significantly in normal segments only (37.2 ± 14.7 ms; p < 0.0001).

**Conclusions:**

Rest and stress BOLD CMR at 3Tesla proved feasible and differentiated between ischemic, non-ischemic, and normal myocardial segments in a clinical patient population. BOLD CMR during vasodilator stress identified patients with significant CAD.

## Background

Cardiovascular magnetic resonance (CMR) is increasingly applied in clinical routine to determine myocardial perfusion [1-4] using contrast enhanced first-pass perfusion techniques [5-8]. Non-invasive characterization of myocardial microcirculation is thought to reflect myocardial tissue supply much better than mere luminographic detection and quantification of epicardial coronary stenosis, and has been shown to be useful for planning of revascularization procedures and cardiac risk stratification.

Blood Oxygen Level Dependent (BOLD) CMR is based on the paramagnetic properties of deoxyhemoglobin as an endogenous contrast agent with increased deoxyhemoglobin content leading to signal reduction on T2*- or T2-weighted images. Thus, BOLD CMR directly reflects myocardial oxygenation status [9,10]. Coronary artery stenosis leads to poststenotic microvascular dilatation in a compensatory effort to maintain sufficient myocardial oxygen supply [11] and blood-oxygen level dependent imaging has been successfully introduced to determine capillary reserve [12,13].

However, data on myocardial BOLD CMR during vasodilator stress in patients with coronary artery disease (CAD) is limited to experimental studies [13-15] and small patient populations [11,12]. The main challenge was low signal intensity differences between normal and pathologic areas of myocardium at 1.5T, and therefore BOLD CMR at 3T may benefit from the inherently higher signal-to-noise ratio (SNR). Hence, in the present study we determined the ability of BOLD CMR to detect stress induced myocardial ischemia and to differentiate between ischemic, non-ischemic, and normal myocardial segments in a population of patients with suspected or known CAD.

## Materials and methods

### Study Group

Forty-six consecutive patients (34 men; age 65 ± 9 years, range 40 to 81 years) referred for clinically indicated invasive coronary x-ray angiography due to chest pain syndromes were prospectively enrolled. Patients were eligible if they had suspected or known CAD (with or without prior percutaneous revascularization or a history of previous myocardial infarction). Patients with prior coronary surgery or typical contraindications for CMR (e.g. incompatible metallic implants, claustrophobia) and administration of adenosine (asthma, AV-block > grade I) were not considered.

All study participants were instructed to refrain from ß-blockers, antianginal medication, cigarettes, tea and coffee for at least 24 hours prior to CMR. Written informed consent was obtained from all subjects, and the Charité Institutional Review Board approved the study.

### CMR Imaging Protocol

CMR was performed with the patient in the supine position using a 3T whole-body imager (Achieva 3T; Philips, Best, the Netherlands) equipped with a Quasar Dual gradient system (40 mT/m, slew rate 200 T/m/sec). A six element cardiac synergy coil was used for signal reception and cardiac synchronization was done with the use of a Vector-ECG. All acquisitions were performed during short end-expiratory breath-holds. After acquisition of cine standard cardiac geometries for the assessment of left ventricular function a fast-gradient-echo multi-echo sequence for BOLD CMR (3-slices of short axis geometry) was performed. Then, adenosine infusion (140 μg/kg/min; maximal total infusion duration of 6 minutes) was started and the identical BOLD CMR sequence was repeated after at least 3 minutes of adenosine infusion. After termination of adenosine infusion, a bolus of 0.2 mmol/kg of gadolinium-DTPA was administered followed by late gadolinium enhancement (LGE) imaging 10 minutes later in identical short axis geometry with full left ventricular coverage.

### CMR Technique

#### Cine Imaging

Three short axis (apical, mid, and basal short axis views) and three long axis geometries (4-, 2-, and 3-chamber view) were acquired using an electrocardiogram-triggered balanced turbo field echo sequence (echo time 1.9 ms, repetition time 4.0 ms, flip angle 40°, spatial resolution 1.8 × 1.8 × 8 mm) during repetitive end-expiratory breath-holding.

#### BOLD CMR

**Figure 1:**
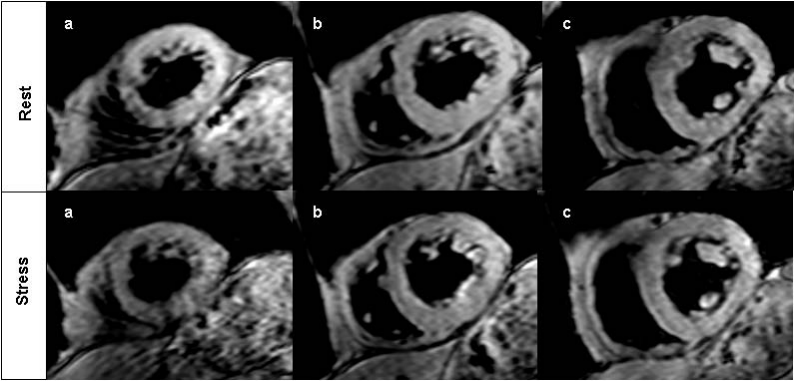
**BOLD CMR at rest (upper row) and during adenosine stress (bottom row) showing the apical (a), midventricular (b) and basal slice (c)**.

BOLD CMR imaging was carried out in the identical three short axis geometries of the left ventricle using an electrocardiogram-triggered, spoiled segmented gradient-echo sequence with six echo times (first echo at 2.7 ms with echo spacing of 1.7 ms, repetition time 13 ms, flip angle 35°, slice thickness 8 mm, reconstructed spatial resolution 1.2 × 1.2 × 8 mm, bandwidth 776 Hz/pixel). Image data acquisition was restricted to end-diastole in order to minimize cardiac motion related artefacts. A black blood dual inversion prepulse was used to nullify signal from flowing blood in the left ventricular cavity. Figure 1 shows a representative example of BOLD CMR at rest and during vasodilator stress.

#### Late Gadolinium Enhancement Imaging

Late gadolinium enhancement imaging was carried out in short axis orientation with full left ventricular coverage using a three-dimensional inversion prepared spoiled gradient echo sequence (echo time 1.8 ms, repetition time 3.7 ms, flip angle 15°; spatial resolution 1.5 × 1.5 × 5 mm). The prepulse delay was determined from an inversion prepared cine scan (Look-Locker) and individually adjusted to optimally suppress signal from normal myocardium.

#### Quantitative Coronary Angiography

All patients underwent invasive coronary angiography in standard Judkins technique within 48 hours after CMR. The procedure was done according to angiographic guidelines using a simultaneous biplane, multidirectional and isocentric x-ray system. At least two orthogonal views of every major coronary vessel and its side branches were acquired. Quantitative coronary angiography (Philips Inturis CardioView, QCA V3.3, Pie Medical Imaging) was performed off-line by an independent observer being unaware of the results of CMR. The severity of coronary stenosis was derived from one single view showing the maximal reduction in absolute luminal diameter and a significant coronary stenosis was defined as ≥50% luminal diameter reduction in vessels with ≥2 mm diameter; significant left main stenosis was considered double-vessel disease. In addition, myocardial segments were assigned to the supplying coronary artery based on a consensus read of the interventionalist and the CMR imager taking the respective coronary dominance type into account.

### Image analysis

#### BOLD CMR

The BOLD CMR data sets were evaluated on a per segment basis according to the standardized 16-segment model [16]. For each segment, the time constant of the signal intensity decay over all echoes was derived. Moreover, the global T2* value per patient averaged over all segmental T2* values was provided at rest and during stress. Image quality of T2* CMR measurements was graded visually on a per patient basis at rest and during stress on a four-point scale as excellent (4 = clear delineation of the left-ventricular myocardium with sharp endocardial contours, no motion artifacts), good (3 = clear delineation of the left-ventricular myocardium but mildly blurred endocardial contours, no motion artifacts), moderate (2 = moderately blurred endocardial contours, occurrence of respiratory/cardiac motion artifacts) or poor (1 = severely reduced delineation of left-ventricular endocardial contours and severe motion artifacts). Occurrence of susceptibility artifacts caused by the heart-lung interface and cardiac veins was scored between 0 and 4 (0 = none, 1 = minor, 2 = moderate, 3 = severe, 4 = non-diagnostic).

#### Late Gadolinium Enhancement Imaging

LGE images were analyzed visually on a per segment basis regarding the presence of myocardial scar ≥25% transmurality with each myocardial segment being classified as normal or scarred.

### Statistical Analysis

Statistical analysis was performed by using the SPSS software package release 17 (Chicago, Ill, USA). The paired Student's t test or Wilcoxon test and independent-samples t test or Mann-Whitney test were used to test for differences within and between groups. All tests were two-tailed. The Kolmogorov-Smirnov test was used to test for normality. A P value of less than 0.05 was considered significant. For ROC analysis and the comparison of the area under the curve Medcalc^® ^release 9.2.1.0. (MedCalc Software, Belgium) was used. The areas under the curves were compared using the method of DeLong et al.[17]

## Results

### Patients Characteristics and Hemodynamic Data

A total of 46 consecutive patients were enrolled in this study. In 4 patients, CMR scans could not be completed: 2 patients were claustrophobic, and 2 did not tolerate adenosine. Hence, 42 (91%) of 46 patients of this study cohort successfully completed the CMR examination and were included in the final analysis.Patient characteristics and hemodynamic data are summarized in table 1 and 2, respectively. Significant CAD was found in 23 patients (54.8%) with ten patients (24%) having single-vessel and 13 patients (31%) multi-vessel disease. According to the results of QCA and LGE imaging a total of 672 myocardial segments were classified as follows: 272 (40.5%) normal segments, 224 (33.3%) non-ischemic segments, 152 (22.6%) ischemic segments. In addition, 24 (3.6%) scarred segments were excluded from further analysis.

**Table 1:** Patient Characteristics

General parameters	
Sex, F/M (%)	12 (29%)/30(71%)
Age, y	64 ± 9
Range, y	40-80
BMI, kg/m^2^	27.9 ± 3.0
	
**Historical information, n(%)**	
Hypertension	36 (86%)
Diabetes mellitus	10 (24%)
Hyperlipoproteinemia	34 (81%)
History of smoking	7 (17%)
CAD in family	10 (24%)
Suspected CAD	30 (71%)
Know CAD	12 (29%)
Previous PCI	10 (24%)
Previous myocardial infarction	7 (17%)
	
**Medication, n (%)**	
ACE inhibitor	31 (74%)
Beta-blocker	33 (79%)
Calcium-channel blocker	8 (19%)
Statins	35 (83%)
	
**Vessel disease, n (%)**	
Single	10 (24%)
Double	8 (19%)
Triple	5 (12%)
Multi	13 (31%)
Left anterior descending coronary artery	17 (40%)
Left circumflex coronary artery	14 (33%)
Right coronary artery	10 (24%)
Without CAD	17 (40%)
Known CAD without Stenosis (≥50%)	2 (5%)

**Table 2:** Left Ventricular Function at rest and Hemodynamic Data

Left ventricular function	
LVEF, %	56 ± 8
LVEDV, mL	151 ± 47
LVESV, mL	71 ± 30
	
**Heart rate, bpm**	
at rest	66 ± 11
max. Stress	83 ± 15*
	
**Systolic blood pressure, mmHg**	
at rest	134 ± 14
max. Stress	132 ± 17
	
**Heart rate-pressure product, bpm × mmHg**	
at rest	8874 ± 1986
max. Stress	11069 ± 2653*

*p < 0.01 rest vs stress

### Image Quality

**Figure 2:**
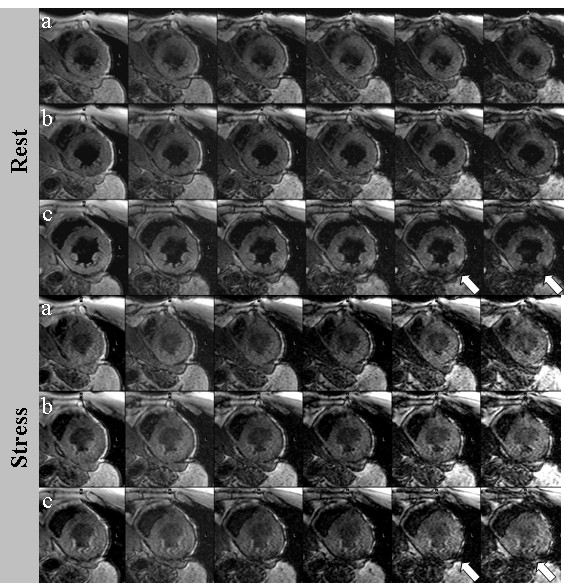
**Example of rest (top rows) and stress (bottom rows) BOLD CMR showing the apical (a), mid-ventricular (b) and basal slice (c) with six echo times**. Susceptibility artifacts (white arrow) occurred at long echo times predominantly in the inferolateral segment caused by the heart-lung interface.

On BOLD CMR at rest, 15 patients showed excellent (35.7%), 21 patients good (50.0%), five patients moderate (11.9%) and one patient poor image quality (2.4%); under adenosine stress image quality was excellent in 11 patients (26,2%), good in 21 patients (50.0%), moderate in eight patients (19.0%) and poor in two patients (4.8%). The mean visual score of BOLD CMR was 3.2 ± 0.8 at rest and 3.0 ± 0.8 under adenosine stress (p = 0.005). Though all studies were of sufficient quality for analysis, segments demonstrating poor image quality were excluded from analysis (17 of 648, 2.6%). The overall artifact score was significantly higher under adenosine stress than at rest (1.6 ± 0.6 vs 1.4 ± 0.5; p = 0.047; for an imaging example see figure 2).

### BOLD CMR

**Figure 3:**
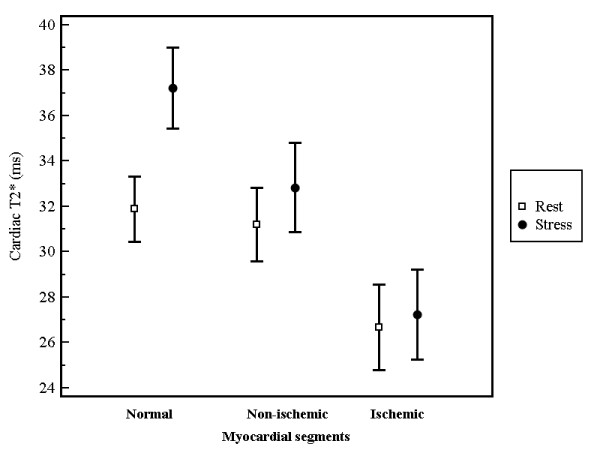
Error bar charts of T2* at rest and under adenosine stress comparing the mean values and corresponding 95% confidence intervals.

**Figure 4:**
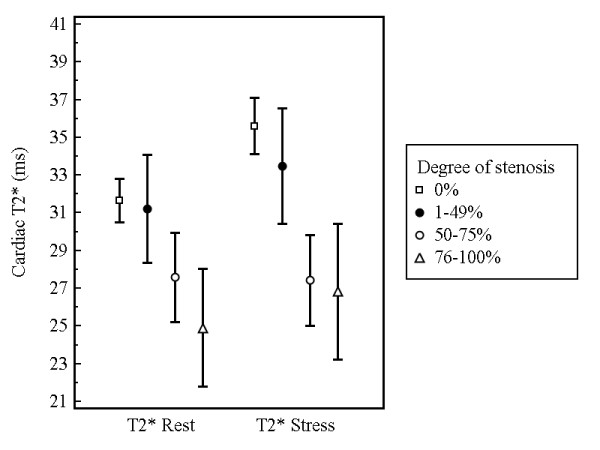
Relationship between T2* values (mean values and corresponding 95% confidence intervals) at rest and under adenosine stress and the degree of coronary artery stenosis.

BOLD measurements at rest revealed significantly lower values for ischemic segments (n = 148; 26.7 ± 11.6 ms) compared to normal segments (n = 260; 31.9 ± 11.9 ms; p < 0.0001) and non-ischemic segments (n = 223; 31.2 ± 12.2 ms; p = 0.0003). During adenosine stress T2* values demonstrated a significant increase in normal segments only (37.2 ± 14.7 ms; p < 0.0001). On the contrary, T2* values of non-ischemic (32.8 ± 14.9 ms; p = 0.19) and ischemic segments (27.2 ± 12.3; p = 0.06) did not significantly differ for the comparison of stress versus rest. When comparing stress T2* values of normal versus non-ischemic and ischemic segments significant differences were found (p = 0.0007 and p < 0.0001, respectively). Corresponding error bar charts are given in figure 3. Figure 4 shows the relationship between the changes of T2* values at rest and under adenosine stress and the degree of coronary artery stenosis.

**Figure 5:**
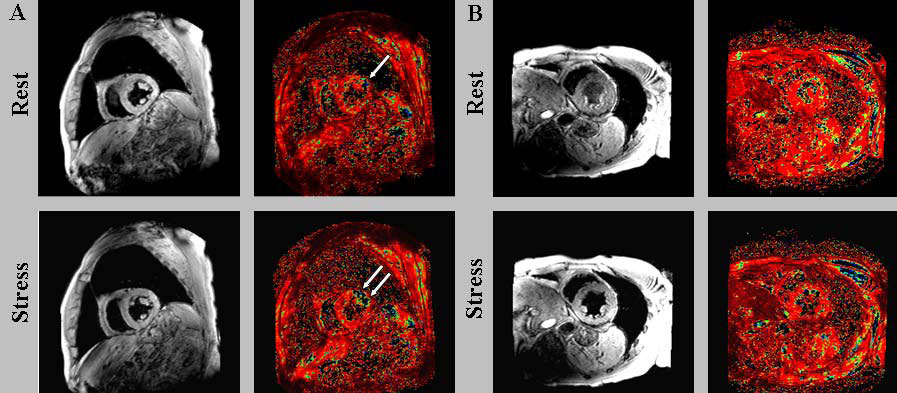
**Imaging examples: Rest and stress BOLD CMR showing a patient with lateral ischemia (A) and a patient without coronary artery disease (B)**.

The global T2* value of patients without significant CAD was 32.2 ± 3.5 ms at rest and 37.4 ± 6.3 ms under stress (p = 0.0003). In patients with significant CAD the global T2* value was 30.3 ± 5.3 at rest and 30.7 ± 5.8 under stress (p = 0.78). Imaging examples are provided in figure 5.

### Diagnostic Performance of BOLD CMR

**Figure 6:**
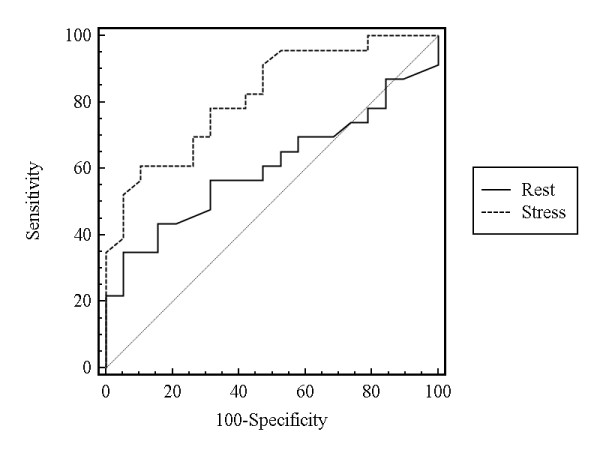
**ROC analysis showing the diagnostic performance of rest and stress BOLD CMR to identify the presence of significant coronary artery disease**.

The area under the curve (AUC) of the ROC analysis (Figure 6) for the ability of T2* CMR imaging to detect significant CAD on a per patient basis differed significantly between rest (0.61, 95%-CI [0.44-0.75]) and stress imaging (0.82, 95%-CI [0.67-0.92], p = 0.015). Using a cut-off value of 33.8 ms, sensitivity and specificity for rest and stress were 78% and 21%, and 78% and 68%. AUC of the ROC analysis for the detection of patients with multivessel disease (≥2 diseased vessels) under stress was 0.80 (95% CI 0.63-0.92). Using a cut-off value 33.8 ms, sensitivity and specificity were 77% and 68%.

## Discussion

In the present study, BOLD CMR at 3T was shown to be feasible at 3T resulting in an overall good image quality at rest and during vasodilator stress. BOLD CMR at rest and under stress conditions distinguished between normal, ischemic and non-ischemic myocardial segments. In addition, adenosine stress BOLD CMR gave a good diagnostic performance with regard to the detection of angiographically defined coronary stenosis.

The non-invasive detection of ischemic myocardium is one of the major strengths of CMR. BOLD imaging represents a promising alternative to first-pass CMR perfusion techniques, which rely on the application of exogenous contrast agents. BOLD imaging allows to directly assess the myocardial oxygenation status taking advantage of the paramagnetic properties of intravascular deoxyhemoglobin [11]. During adenosine stress, myocardial segments being supplied by normal coronary arteries show an increased oxygen content while deoxyhemoglobin concentration is low resulting in a higher signal on T2*- or T2-weighted images. On the contrary, myocardial segments being supplied by a stenosed coronary artery exhibit a dilated capillary bed already under resting conditions in order to maintain sufficient blood supply. Hence, additional vasodilatation under stress is minimal and the hyperaemic response is abolished.

In the present study, ischemic myocardial segments showed significantly reduced T2* values under resting conditions, while during adenosine stress T2* values increased in normal myocardial segments only. Our findings were consistent with previous reports by Wacker et al. and Niemi et al. [11,18], but somewhat contrasted the results by Friedrich et al. [12] even demonstrating a signal decrease during vasodilator stress in myocardial territories supplied by coronary arteries with >75% luminal narrowing.

Interestingly, in the present study non-ischemic myocardial segments did not show a significant increase of T2* values during stress. These findings may be explained by microvascular disease [19], which is present in myocardial segments of patients with CAD despite the absence of significant epicardial coronary artery narrowing. The T2* measurements of the current study showed a relation between the degree of coronary stenosis and the T2* value at rest and under adenosine stress.

The T2* CMR values at rest in patients without CAD were within the range of previously published data by Story et al. (33.3 ± 8.3 ms, outliers were excluded) [20] and O'Regan et al. (27.3 ± 6.4 ms) [21] using a single-slice image acquisition. However, in our study, the standard deviation of T2* values was substantially larger, which can be explained by the imaging strategy employing a 3-slice acquisition per patient in order to achieve coverage of all 16-standard myocardial segments. Susceptibility artifacts appeared predominantly in the inferolateral segments of the myocardium on images with the longest echo times and resulted mainly from interference with cardiac veins [22] and the heart-lung interface [23]. In order to reduce these artifacts an end-expiratory breath hold was used as proposed by Wacker et al.[9] in conjunction with a black blood prepulse to reduce possible sources of artifacts arising from the blood pool. Furthermore, the use of only six echo times and a relatively short echo spacing and repletion time of 13 ms in our study prevents susceptibility artefacts caused by longer echo times [9]. Recently, O'Regan et al. [21] reported that black blood breath-hold multiecho T2* imaging can be adequately performed at 3T and resulted in only minor susceptibility artifacts in the inferolateral region of the heart.

Importantly, the current study population consisted of consecutive patients with a high proportion of known CAD and prior myocardial infarctions but such patients were usually not considered in previous studies [11,12,21]. However, a direct comparison to first pass CMR perfusion or SPECT imaging would have been desirable and may be considered a limitation of the present study.

## Conclusions

Rest and stress BOLD CMR at 3T proved feasible and may be used to differentiate between ischemic, non-ischemic, and normal myocardial segments in a clinical patient population. BOLD CMR during vasodilator stress identified patients with significant CAD.

## Authors' contributions

RM: study design, data acquisition, image analysis, statistical analysis, manuscript drafting. IP: study design, image analysis, statistical analysis, manuscript drafting. BS: study design, literature research, manuscript drafting. EF: study design, manuscript drafting. RG: data acquisition, literature research. CJ: study design, statistical analysis, manuscript drafting, guarantator of integrity of entire study. All authors have made revisions to the manuscript and have read and approved the final version.

## Competing interests

Bernhard Schnackenburg is an employee of Philips Medical Systems, Hamburg, Germany.

## List of abbreviations

CMR: Cardiovascular magnetic resonance; CAD: Coronary artery disease; LGE: Late gadolinium enhancement; SNR: Signal-to-noise ratio; QCA: Quantitative coronary angiography; ROC: Receiver under the operator curve; BMI: Body mass index; PCI: Percutaneous coronary intervention.

## References

[B1] Panting JR Gatehouse PD Yang GZ Jerosch-Herold M Wilke N Firmin DN Pennell DJ Echo-planar magnetic resonance myocardial perfusion imaging: parametric map analysis and comparison with thallium SPECT J Magn Reson Imaging 2001 13 192 200 10.1002/1522-2586(200102)13:2<192::AID-JMRI1029>3.0.CO;2-N 11169824

[B2] Schwitter J Nanz D Kneifel S Bertschinger K Buchi M Knusel PR Marincek B Luscher TF von Schulthess GK Assessment of myocardial perfusion in coronary artery disease by magnetic resonance: a comparison with positron emission tomography and coronary angiography Circulation 2001 103 2230 2235 11342469 10.1161/01.cir.103.18.2230

[B3] Schwitter J Wacker CM van Rossum AC Lombardi M Al-Saadi N Ahlstrom H Dill T Larsson HB Flamm SD Marquardt M Johansson L MR-IMPACT: comparison of perfusion-cardiac magnetic resonance with single-photon emission computed tomography for the detection of coronary artery disease in a multicentre, multivendor, randomized trial Eur Heart J 2008 29 480 489 10.1093/eurheartj/ehm617 18208849

[B4] Kelle S Graf K Dreysse S Schnackenburg B Fleck E Klein C Evaluation of contrast wash-in and peak enhancement in adenosine first pass perfusion CMR in patients post bypass surgery J Cardiovasc Magn Reson 2010 12 28 10.1186/1532-429X-12-28 20465836 PMC2887852

[B5] Al-Saadi N Nagel E Gross M Bornstedt A Schnackenburg B Klein C Klimek W Oswald H Fleck E Noninvasive detection of myocardial ischemia from perfusion reserve based on cardiovascular magnetic resonance Circulation 2000 101 1379 1383 10736280 10.1161/01.cir.101.12.1379

[B6] Nagel E Klein C Paetsch I Hettwer S Schnackenburg B Wegscheider K Fleck E Magnetic resonance perfusion measurements for the noninvasive detection of coronary artery disease Circulation 2003 108 432 437 10.1161/01.CIR.0000080915.35024.A9 12860910

[B7] Wilke N Jerosch-Herold M Wang Y Huang Y Christensen BV Stillman AE Ugurbil K McDonald K Wilson RF Myocardial perfusion reserve: assessment with multisection, quantitative, first-pass MR imaging Radiology 1997 204 373 384 9240523 10.1148/radiology.204.2.9240523

[B8] Gebker R Jahnke C Paetsch I Kelle S Schnackenburg B Fleck E Nagel E Diagnostic performance of myocardial perfusion MR at 3 T in patients with coronary artery disease Radiology 2008 247 57 63 10.1148/radiol.2471070596 18305188

[B9] Wacker CM Bock M Hartlep AW Beck G van Kaick G Ertl G Bauer WR Schad LR Changes in myocardial oxygenation and perfusion under pharmacological stress with dipyridamole: assessment using T*2 and T1 measurements Magn Reson Med 1999 41 686 695 10.1002/(SICI)1522-2594(199904)41:4<686::AID-MRM6>3.0.CO;2-9 10332843

[B10] Bauer WR Nadler W Bock M Schad LR Wacker C Hartlep A Ertl G The relationship between the BOLD-induced T(2) and T(2)(*): a theoretical approach for the vasculature of myocardium Magn Reson Med 1999 42 1004 1010 10.1002/(SICI)1522-2594(199912)42:6<1004::AID-MRM2>3.0.CO;2-M 10571919

[B11] Wacker CM Hartlep AW Pfleger S Schad LR Ertl G Bauer WR Susceptibility-sensitive magnetic resonance imaging detects human myocardium supplied by a stenotic coronary artery without a contrast agent J Am Coll Cardiol 2003 41 834 840 10.1016/S0735-1097(02)02931-5 12628730

[B12] Friedrich MG Niendorf T Schulz-Menger J Gross CM Dietz R Blood oxygen level-dependent magnetic resonance imaging in patients with stress-induced angina Circulation 2003 108 2219 2223 10.1161/01.CIR.0000095271.08248.EA 14557359

[B13] Shea SM Fieno DS Schirf BE Bi X Huang J Omary RA Li D T2-prepared steady-state free precession blood oxygen level-dependent MR imaging of myocardial perfusion in a dog stenosis model Radiology 2005 236 503 509 10.1148/radiol.2362040149 16040907

[B14] Fieno DS Shea SM Li Y Harris KR Finn JP Li D Myocardial perfusion imaging based on the blood oxygen level-dependent effect using T2-prepared steady-state free-precession magnetic resonance imaging Circulation 2004 110 1284 1290 10.1161/01.CIR.0000140673.13057.34 15326062

[B15] Zhang H Gropler RJ Li D Zheng J Assessment of myocardial oxygen extraction fraction and perfusion reserve with BOLD imaging in a canine model with coronary artery stenosis J Magn Reson Imaging 2007 26 72 79 10.1002/jmri.20964 17659557

[B16] Cerqueira MD Weissman NJ Dilsizian V Jacobs AK Kaul S Laskey WK Pennell DJ Rumberger JA Ryan T Verani MS Standardized myocardial segmentation and nomenclature for tomographic imaging of the heart: a statement for healthcare professionals from the Cardiac Imaging Committee of the Council on Clinical Cardiology of the American Heart Association Circulation 2002 105 539 542 10.1161/hc0402.102975 11815441

[B17] DeLong ER DeLong DM Clarke-Pearson DL Comparing the areas under two or more correlated receiver operating characteristic curves: a nonparametric approach Biometrics 1988 44 837 845 10.2307/2531595 3203132

[B18] Niemi P Poncelet BP Kwong KK Weisskoff RM Rosen BR Brady TJ Kantor HL Myocardial intensity changes associated with flow stimulation in blood oxygenation sensitive magnetic resonance imaging Magn Reson Med 1996 36 78 82 10.1002/mrm.1910360114 8795024

[B19] Maseri A Crea F Kaski JC Crake T Mechanisms of angina pectoris in syndrome X J Am Coll Cardiol 1991 17 499 506 10.1016/S0735-1097(10)80122-6 1991909

[B20] Storey P Thompson AA Carqueville CL Wood JC de Freitas RA Rigsby CK R2* imaging of transfusional iron burden at 3T and comparison with 1.5T J Magn Reson Imaging 2007 25 540 547 10.1002/jmri.20816 17326089 PMC2884049

[B21] O'Regan DP Callaghan MF Fitzpatrick J Naoumova RP Hajnal JV Schmitz SA Cardiac T2* and lipid measurement at 3.0 T-initial experience Eur Radiol 2008 18 800 805 10.1007/s00330-007-0814-8 18034347

[B22] Reeder SB Faranesh AZ Boxerman JL McVeigh ER In vivo measurement of T*2 and field inhomogeneity maps in the human heart at 1.5 T Magn Reson Med 1998 39 988 998 10.1002/mrm.1910390617 9621923 PMC2396319

[B23] Atalay MK Poncelet BP Kantor HL Brady TJ Weisskoff RM Cardiac susceptibility artifacts arising from the heart-lung interface Magn Reson Med 2001 45 341 345 10.1002/1522-2594(200102)45:2<341::AID-MRM1043>3.0.CO;2-Q 11180442

